# Trauma as a Factor in Appendicitis

**Published:** 1916-04

**Authors:** Lawrence Geo. Hanley

**Affiliations:** Buffalo, N. Y.


### BUFFALO MEDICAL JOURNAL

Yearly Volume 71 APRIL, 1916 No. 9

### ORIGINAL ARTICLES

**The right is reserved to decline papers not dealing with practical medical and surgical subjects, and such as might offend or fail to interest readers. Contributors are solely responsible for opinions, methods of expression and revision of proof.**

### Trauma as a Factor in Appendicitis.

By LAWRENCE GEO. HANLEY, M.D., LL.D., Buffalo, N. Y.

"There is one quality the possession of which is the supreme need of the physician, without which he is as unfit as a tone-deaf musician, or a color-blind painter; that is the faculty of exact observation."—Minot, late professor of Harvard.

Appendicitis is a common malady and people continue to die from it, chiefly because they insist upon postponing an operation, although there is no doubt that operation is the only safe treatment. With prompt operation the mortality is insignificant, but without it the death rate may perhaps be as high as 20 per cent.

Deaths from appendicitis are decreasing practically every year because intelligent people are learning that prompt surgical aid is the only rational procedure, and that what causes death even in neglected cases is almost invariably the appendicitis, not the operation.

One reason, however, why some patients oppose surgical measures is the inability of the attending physician, or surgeon, as a rule, to specify the cause of the disorder, and this defect on our part is a direct result of the absence of the faculty of exact observation among medical men as a body—using the words "exact observation" in their widest sense.

Unless we question our patients who suffer from appendicitis and, in addition, study the history of each case, the etiology of the malady would remain obscure as in very many instances today.

It is my opinion that trauma plays a far greater part than is generally supposed, and in procuring the history—trauma has been given very little attention—satisfied that it is a case of appendicitis, operate, exit appendix.

Injury to the right iliac region certainly lessens the tone of the structures in that area, the same as in any other part and when lowered beyond a certain grade a locus minoris resistentiae is formed, and the blood that bathes that section carries with it bacteria or else gives the bacteria already existing there a chance to become active. The existence of the effects of some previous inflammatory condition, or the presence of a coprolith, may be tardily or suddenly set into inflammatory action by blows, kicks, straining, lifting, jumping, or other traumatic causes.

It is not my intention to enter too deeply in detail regarding the pathology, but only to report a few cases from trauma that have come under my notice, trusting that more light may be thrown upon this important phase of the subject.

Would we be justified in saying that jumping from a street car, straining, lifting, bicycle riding—not to mention a direct blow over the region of the appendix—was the prime means of producing disease in that organ? Yet there are cases as such reported by men who are masters in their line. One operator says that trauma is a direct factor in the causation of some cases more often than has been supposed, and reports some fifty cases collected by him. In The Medical Record of June 2, 1906, there is a report of three cases due to trauma.

Another operator remarks: "In a careful research of histories of 500 cases of acute appendicitis there was no evidence of traumatism as a causative factor, and in my whole experience I have never encountered an undoubted instance of appendicitis caused by trauma."

John E., age 35, fireman. Family history negative. No history of injury or previous sickness until Jan. 16th, 1911, when he fell from a ladder a distance of fifteen feet. He struck upon his abdomen and, aside from a slight shock, he experienced no great discomfort, and he returned to his work and continued for three days. Four days after the accident he was taken with sharp pains in abdomen, cramps, vomiting, pain localized in region of appendix, board-like belly; temperature 99, pulse 120. Five days after accident temperature 100, pulse 110, increased pain. Removed to Sisters' Hospital. Operation. An appendix four inches long, one-third of an inch in thickness imbedded in a fold of discolored omentum was removed. He made an easy recovery.

Joseph C., age 32. Street car conductor. Operated May 24th, 1914. Was standing near entrance of car when he was thrown against angle of iron rail, result of a collision. He was hurt in the abdomen. Complained of pain for one week, and was attended by Dr. S. Moore, who called me in consultation eight days after the accident. Temperature 99, pulse 110, nausea, pain over region of appendix. Operation. Appendix was congested, was about three inches long and lying in a bed of omentum, which was also thickened and inflamed. There was also some escaped sanguinious fluid when appendix was delivered. Nothing intervened and patient made good recovery.

J. C., age 29. Clerk. Family history good. Personal history negative as regards sickness and injury, excepting in 1912 when he strained himself playing handball, and since that time he has had pain at frequent intervals in lower right side of abdomen. He was never obliged to leave his work until Feb. 14, 1914, when he was seized with sharp pain over site of appendix, nausea; temperature normal, pulse 108. Operation was recommended and performed. The appendix was imbedded in caecum and covered with omentum, showing the effects of previous inflammatory action. Satisfactory recovery. Has had no return of former symptoms and was away from his duties but four weeks. He dates his trouble from straining himself in 1912. It is my opinion that trauma was the causative factor of his appendicitis.

John B., age 55. Motorman. Nov. 24th, 1905. Family history good. Personal history nothing of importance. While releasing a brake handle struck him in abdomen, causing him great pain, in his own words "it knocked me wind out." Nevertheless this did not prevent him from working for three days. After that time he was confined to the house for one week. He returned to work and continued for two weeks, when he was obliged to discontinue. He finds relief only when lying on his abdomen, or when leaning forward. Pain is centralized over region of appendix. Operation advised and done. There was an adhesion of appendix and mesentary to the abdominal peritoneum. Appendix removed. Made a good recovery and is free from pain and able to return to his regular duties.

Mr. B. Pullman conductor. August 20th, 1909. Nothing in family or personal history of any moment. Was thrown against a seat by a sudden jolting of a car while rounding a curve. Taken to his home in a carriage. Saw him two days after the injury. Nausea, temperature 100, pulse 100. Pain over region of appendix. August 23d, 1909, sent to hospital. Leucocytosis 17000. Aug. 24th operated. A dead appendix, containing a coprolith. Made a good recovery. He informed me that prior to his injury he was perfectly well. How long that coprolith was in his appendix I know not, but it is my opinion that the injury set up the inflammatory action that necessitated operative interference.

The following cases were reported by me in the Buffalo Medical Journal January, 1906:

Charles Z., age 33. Fireman. Has always been well until three days before operation, date of which was Oct. 13th, 1905. He thinks he might have hurt himself sliding down a pole in firehouse. Pulse 90, temperature 101. Leucocytosis 16000. There is pain upon the slightest pressure and spasm of muscle of right side extending from Poupart ligament to ninth intercostal space. On opening abdomen a piece of omentum 6x6x1 inches thick adherent to peritoneum, and ilium and caecum in a strangulated semi-gangrenous condition was found. The appendix lay posterior to this mass and was greatly inflamed from contact with diseased omentum. The serosa was very much engorged but the mucosa seemed normal. The peritoneum, where the omentum was attached, and the serous surface of ilium, bled freely when separated. Patient made a good recovery. Left hospital on twenty-first day.

John S., age 43. Family history good. Personal history good until one month ago when he strained himself lifting. The pain he experienced at that time did not hinder him from occupying his occupation as a teamster. On Nov. 15th, 1905, came under my care. Examination shows localized spasm of muscles extending from Pouparts ligament to median line and ninth intercostal space. Painful upon pressure and so severe that patient is unable to stand, and only finds relief when lying upon his back with lets flexed. Pulse 98, temperature 99. Leucocyte count 8,000. On opening abdomen a piece of omentum 4x3 inches was found adhered to caecum and peritoneum strangulated and semi-gangrenous. The appendix and mass were removed. Patient's condition for first four days was very satisfactory, but on the fifth day he devoloped a delirium the nature of a nimia bibens—though his history showed that he was not in the habit of using intoxicants in excess, and in the absence of the nurse jumped from the third story window, breaking glass, and fell a distance of forty feet. Though he broke the glass he did not break any bones, and from a few cuts on his face and bruises on his body he made a good recovery. Wound and abdomen healed and he left hospital in 20 days.

That trauma is a factor in producing appendicitis is my opinion. That it is doubted by some is also true, and that it is of little importance as regards the treatment is correct, but from a medico-legal standpoint demands great consideration.

"To know a subject thoroughly has never been given to erring, sinful man," but it is our duty to try and acquire that knowledge, and I believe that if we question our patient's suffering from appendicitis we will find that trauma is a causative factor more often than we thought possible.

428 Porter Avenue.

---

**Early Recognition of Cancer of Stomach.** Julius Friedenwalk, Baltimore, Md. Med. Jour., Feb. The ordinary symptoms are mentioned, with percentage of occurrence. These, of course, are well known and only of suggestive value. Haematemesis occurred in 22.7% but early enough for diagnosis to be practical, in only about 4%. In nearly 90% of the cases in which haematemesis occurred at all, it was repeated. Melaena occurred in 18.9% but early in only about 3%. Occult blood occurred in 92.5%, was usually constant if once observed, and usually occurred early. (Note: In other words, if blood is to be sought at all, it should be looked for where it will occur without abnormal reflexes, i.e. in the stools, and it should be looked for carefully, i.e. by chemic test and not by naked-eye observation. Ed.) Dysphagia occurred in 78% of involvements of the cardia and occurred early. (Yet we have recently seen a case of marked obstruction to the tube, verified by operation, with no subjective complaint of dysphagia. Ed.) Palpable tumor was a late sign. Gastric dilatation due to pyloric stenosis occurred in 47%, in fact in 52% of early cases. X-ray findings are stated to be valuable, without statistics. Filling defect is especially emphasized. Absence of HCl occurred in 89%, in 76% as an early sign. Oppler-Boas bacilli were found in 79%, early in 74%, of course only when lactic acid was present. (Note: The mere absence of HCl is not of much significance because it is likely to be noted in non-malignant conditions, especially in the age period at which cancer is most prevalent. Ed.) The Wolff-Junghans test occurred in 84.6% (44 of 52 cases) and early in 79.5% (11 of 14 cases) and is regarded as very valuable. The Abderhalden and Kelling serum tests were not found to be specific, variations occurring in both directions.

---

**Brain Lipoid as a Haemostatic.** Arthur D. Hirschfelder, Lancet, Sept. 4, 1915. Ox brain is first washed with alcohol. The residue is extracted with ether, filtered and evaporated. A yellow residue is obtained consisting mainly of kephalin, which produces haemostatsis on being smeared over a bleeding surface. On account of its cheapness, it is especially applicable to military surgery.

